# Drug delivery strategies for neuroprotective therapy in ischemic stroke: Application of nanotechnology

**DOI:** 10.4103/NRR.NRR-D-24-01383

**Published:** 2025-05-06

**Authors:** Zhan Jiang, Qi Chen, Huanghao Yang

**Affiliations:** 1 Fujian Engineering Research Center for Intelligent Health Diagnostic Technology, Interdisciplinary Institute for Medical Engineering, Fuzhou University, Fuzhou, Fujian Province, China; 2 New Cornerstone Science Laboratory, MOE Key Laboratory for Analytical Science of Food Safety and Biology, College of Chemistry, Fuzhou University, Fuzhou, Fujian Province, China

**Keywords:** drug delivery, excitotoxicity, ischemic stroke, ischemia-reperfusion, nanoparticles, nerve regeneration, neuroinflammation, neuroprotection, oxidative stress, pathophysiology

## Abstract

The mechanisms underlying the pathophysiology of ischemic stroke are complex and multifactorial and include excitotoxicity, oxidative stress, inflammatory responses, and blood–brain barrier disruption. While vascular recanalization treatments such as thrombolysis and mechanical thrombectomy have achieved some success, reperfusion injury remains a significant contributor to the exacerbation of brain injury. This emphasizes the need for developing neuroprotective strategies to mitigate this type of injury. The purpose of this review was to examine the application of nanotechnology in the treatment of ischemic stroke, covering research progress in nanoparticle-based drug delivery, targeted therapy, and antioxidant and anti-inflammatory applications. Nano-based drug delivery systems offer several advantages compared to traditional therapies, including enhanced blood–brain barrier penetration, prolonged drug circulation time, improved drug stability, and targeted delivery. For example, inorganic nanoparticles, such as those based on CeO_2_, have been widely studied for their strong antioxidant capabilities. Biomimetic nanoparticles, such as those coated with cell membranes, have garnered significant attention owing to their excellent biocompatibility and targeting abilities. Nanoparticles can be used to deliver a wide range of neuroprotective agents, such as antioxidants (e.g., edaravone), anti-inflammatory drugs (e.g., curcumin), and neurotrophic factors. Nanotechnology significantly enhances the efficacy of these drugs while minimizing adverse reactions. Although nanotechnology has demonstrated great potential in animal studies, its clinical application still faces several challenges, including the long-term safety of nanoparticles, the feasibility of large-scale production, quality control, and the ability to predict therapeutic effects in humans. In summary, nanotechnology holds significant promise for the treatment of ischemic stroke. Future research should focus on further exploring the mechanisms of action of nanoparticles, developing multifunctional nanoparticles, and validating their safety and efficacy through rigorous clinical trials. Moreover, interdisciplinary collaboration is essential for advancing the use of nanotechnology in stroke treatment.

## Introduction

Ischemic stroke has become a significant global health issue, ranking as the leading cause of disability and the second leading cause of death worldwide (Campbell et al., 2019; Li et al., 2025; Yu et al., 2025). Cerebral ischemia reduces the availability of glucose and oxygen in the brain, resulting in an energy deficit for neurons, endothelial cells, and glial cell (Zhou et al., 2025; Zhu et al., 2025). This energy deficiency progresses to anoxic depolarization and decreased glutamate reuptake, leading to cellular excitotoxicity, blood–brain barrier (BBB) dysfunction, and the release of signaling molecules, such as cytokines, from microglia, oligodendrocytes, and astrocytes. The primary management strategy for ischemic stroke involves rapid reperfusion therapies, such as intravenous thrombolysis and endovascular clot retrieval, which can significantly reduce disability if administered promptly (Tuo et al., 2022).

For thrombolytic therapy, recombinant tissue-type plasminogen activator (rt-PA) is a clinically common thrombolytic agent, including alteplase and tenecteplase. While rt-PA remains the Food and Drug Administration-approved thrombolytic agent for intravenous thrombolysis in acute ischemic stroke (Lee et al., 2018), its therapeutic application usually carries the risk of reperfusion injury, a paradoxical complication characterized by oxidative stress cascades and subsequent neuroinflammatory responses that may exacerbate cerebral damage (Tiedt et al., 2022). To mitigate reperfusion injury in the treatment of ischemic stroke, research has focused on the development of neuroprotective agents, such as ferroptosis inhibitors and antioxidants (Briones-Valdivieso et al., 2024; Tian et al., 2024). However, their effectiveness is often limited by their low biocompatibility and inability to cross the BBB. The BBB plays a crucial role in maintaining the microenvironment of the brain, allowing only specific substances, such as oxygen, carbon dioxide, and glucose, to enter the brain parenchyma (Qiao et al., 2024). Most drugs and proteins have large molecular sizes, which limits their ability to penetrate the BBB (Ding et al., 2020; He et al., 2021b). Consequently, there is an urgent need to develop drug-delivery systems that can effectively transport medications into the brain.

Nano-based drug delivery systems are being increasingly used in the treatment of ischemic stroke. The modification of these systems with targeted functional groups allows precise drug targeting, which reduces adverse effects on surrounding tissues. These systems also facilitate the sustained release of drugs, prolonging their residence time in the body and enhancing their effectiveness. This can lead to a reduction in the required dosage and a lower risk of toxicity (Wu et al., 2024). Furthermore, nano-based systems can integrate multiple therapeutic functions, such as anti-inflammatory and antioxidant activities, which enhance their therapeutic efficacy (Yang et al., 2024). Inorganic nanoparticles (NPs), such as fullerene, CeO_2_, Mn_3_O_4_, and heteropolyacid NPs, are particularly effective in reducing oxidative stress (ROS) owing to their notable ability to scavenge ROS.

Polymeric NPs, including polyethylene glycol and PLGA NPs, have been shown to enhance the solubility, stability, and transmembrane transport of drugs, thereby improving their biocompatibility and bioavailability (Wang et al., 2023; Yan et al., 2023a). In addition to serving as excellent drug carriers, certain polymers, such as polydopamine, can reduce ROS availability, which helps alleviate oxidative stress (Yin et al., 2024; Jian et al., 2025). Lipid nanoparticles (LNPs), composed of biodegradable and biocompatible amphiphiles such as fatty acids and phospholipids, can be surface-modified with targeting ligands to enhance their targeting capabilities (Nong et al., 2024). Biomimetic NPs, including those coated with cell membranes and extracellular vesicles (EVs), have natural properties that enable them to cross the BBB and target ischemic regions and also display excellent biocompatibility and bioavailability (Hirsch et al., 2023). In summary, these beneficial properties endow NPs with distinct advantages over traditional small-molecule drugs.

In this review, we first provide a brief overview of the mechanisms underlying the pathophysiology of ischemic stroke. Subsequently, we summarize related therapies, focusing on recent advancements in NP applications in this field, and offer perspectives on future research directions. We discuss the potential of nano-based drug delivery systems to inhibit inflammation, reverse neuronal damage, and protect nerve cells. Additionally, we explore different delivery routes and strategies, highlighting the unique advantages of NPs over traditional drugs in the treatment of ischemic stroke. Furthermore, we outline the different types of NPs employed in stroke therapy and examine the challenges associated with their preclinical applications, while also emphasizing their potential for clinical translation. This work serves as a reference for the development of innovative NP-based therapies for ischemic stroke.

## Search Strategy

**Figure 1:**
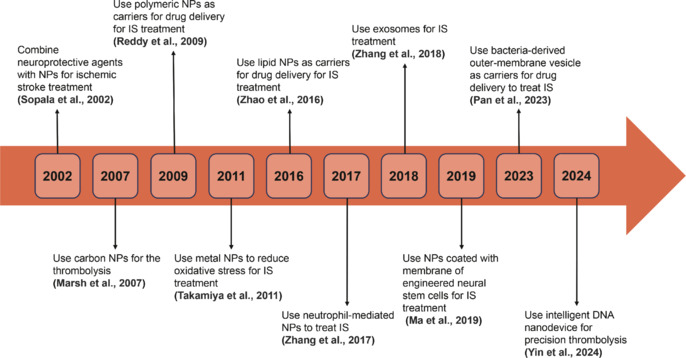
Timeline of research development of NPs for the treatment of IS. IS: Ischemic stroke; NPs: nanoparticles.

In this review, articles published between September 2019 and September 2024 that met the search criteria were retrieved from the PubMed database. A refined search strategy, using keywords and terms such as “ischemic stroke” and “nanoparticle,” yielded 311 relevant documents specifically discussing the use of NPs in the treatment of ischemic stroke. The search was conducted in September 2014. Significant milestones in this area are illustrated in **Figure 1**.

## Pathophysiology of Ischemic Stroke and Relevant Drugs for Its Treatment

Most cases of ischemic stroke are thromboembolic in nature and commonly originate from large artery atherosclerosis and cardiovascular diseases, particularly atrial fibrillation. Atheroembolic ischemic stroke occurs when atheromatous plaques in the neck, head arteries, or ascending aorta rupture, releasing cholesterol elements that can embolize into the cerebral arteries. This type of stroke is more common in patients with elevated levels of low-density lipoprotein and low levels of high-density lipoprotein (Jia et al., 2024). Notably, ischemic strokes resulting from small vessel disease, which are associated with diabetes and high blood pressure, are particularly prevalent in Asia (Campbell et al., 2019).

Shortly after ischemia, blood flow rapidly decreases within the ischemic core, leading to hypoperfusion, which limits the supply of oxygen and glucose to the brain. As oxygen and glucose levels decline in the ischemic brain tissue, the synthesis of high-energy phosphate compounds, particularly adenosine triphosphate, is impaired. This impairment disrupts energy-dependent physiological activities, such as ion transport, which are essential for cellular viability. It also triggers numerous downstream processes that lead to cell death (apoptosis, autophagy, ferroptosis, and necroptosis), ultimately forming the infarct area (Moskowitz et al., 2010). During the reperfusion phase, the restoration of blood flow—while necessary for recovery—paradoxically aggravates damage by increasing the generation of reactive nitrogen species (RNS) and ROS. The resulting oxidative stress damages the neurovascular units, exacerbates cell death, and stimulates the recruitment of microglia and the expression of pro-inflammatory mediators (Sun et al., 2018). This process leads to the infiltration of macrophages and neutrophils, which further disrupts the BBB and contributes to brain edema (Huang et al., 2006).

### Cell excitotoxicity

**Figure 2:**
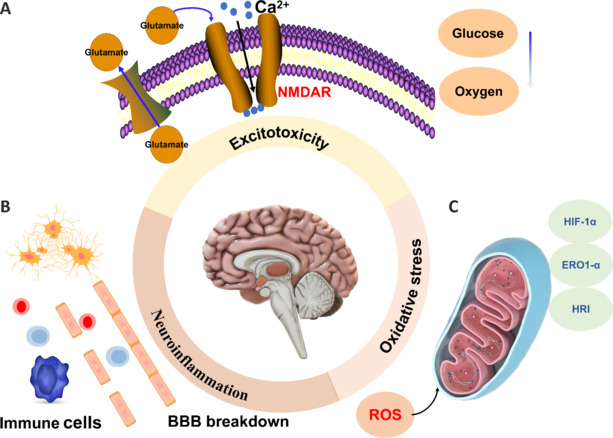
Summary of the molecular mechanisms involved in the pathophysiology of ischemic stroke. The common pathophysiology of ischemic stroke includes excitotoxicity, neuroinflammation, and oxidative stress. (A) Excitotoxicity. (B) Neuroinflammation. (C) Oxidative stress. BBB: Blood–brain barrier; ERO1-α: endoplasmic reticulum disulfideoxidase 1-α; HIF-1α: hypoxia-inducible factor 1 alpha; HRI: heme-regulated inhibitor; NMDAR: N-methyl-D-aspartate receptor; ROS: reactive oxygen species.

The excessive release of the excitatory neurotransmitter glutamate is a major contributor to neuronal injury (**Figure 2A**). Under normal conditions, extracellular glutamate concentrations are kept low through the action of specific transporter proteins, in a manner that relies on transmembrane electrochemical ion gradients, primarily sodium (Na^+^). However, during a stroke, the deprivation of oxygen and glucose impairs the activity of the ion pumps responsible for sustaining these gradients, thereby disrupting them and causing glutamate transporters to secrete glutamate into the extracellular environment rather than uptake it. The excessive binding of extracellular glutamate to receptors on nerve cells, particularly N-methyl-D-aspartate receptors (NMDARs), leads to an over-influx of calcium ions. This influx promotes enzymatic activities that lead to the degradation of cellular constituents, including lipids, proteins, and nucleic acids (Choi et al., 1988; Yu et al., 2023). Furthermore, elevated intracellular calcium concentrations can impair mitochondrial function, thus exacerbating energy deficiency and potentially initiating programmed cell death pathways (Kristián and Siesjö, 1996; Rahi and Kaundal, 2024).

Memantine is a non-competitive antagonist of NMDARs that serves as a neuroprotective agent in ischemic stroke. It inhibits the excitotoxic cascade caused by excessive calcium influx through NMDARs, thereby reducing neuronal death and improving functional outcomes after a stroke. Memantine has been demonstrated to provide neuroprotection through multiple mechanisms (Tuo et al., 2022). In animal models, memantine reduces infarct size, inhibits neuronal apoptosis, decreases brain edema, and enhances neurological function. Additionally, the effectiveness of memantine is time-sensitive, with greater benefits observed when it is administered promptly after the onset of ischemia (Seyedsaadat and Kallmes, 2019). Clinical trials have indicated that high-dose memantine can improve scores on the Barthel Index and the NIH Stroke Scale (NIHSS) among stroke patients, suggestive of improved neurological and functional outcomes. However, these clinical studies had several limitations, including small sample sizes and varying study designs, which affected the generalizability of the findings (Berthier et al., 2009; Pichardo-Rojas et al., 2023). Further research is needed to confirm these findings and validate the clinical utility of memantine.

Nimodipine, a selective calcium channel blocker, inhibits the influx of calcium ions, thereby protecting against cell excitotoxicity. Additionally, nimodipine has a high affinity for specific receptors in cerebral vessels, giving it greater selectivity for these vessels compared to other calcium channel blockers (Scriabine and van den Kerckhoff, 1988). Its high lipophilicity allows it to readily cross the BBB. Studies using animal models have demonstrated that nimodipine effectively dilates cerebral vessels and enhances cerebral blood flow (Tomassoni et al., 2008; Sommer et al., 2024; Yang et al., 2024). Furthermore, a recent study showed that nimodipine reduces cerebral edema, enhances mitochondrial function, and prevents BBB breakdown by lowering the levels of intercellular adhesion molecule-1 (ICAM-1) and matrix metalloproteinase (MMP)-9 (Shadman et al., 2024). These findings suggest that nimodipine has potential as a neuroprotective agent through its anti-inflammatory and mitochondrial function-enhancing effects. Moreover, a clinical study indicated that while nimodipine did not significantly prevent cognitive decline, its administration resulted in moderate cognitive improvement, particularly that relating to memory. The safety profile of nimodipine was similar to that of a placebo in the management of cognitive impairment following a stroke (Zheng et al., 2019).

Postsynaptic density protein 95 (PSD-95) serves as a synaptic scaffolding protein, linking NMDARs and nitric oxide synthase (NOS) (Kornau et al., 1995). Nerinetide (NA1) targets PSD-95 and inhibits its interaction with NMDARs, thereby inhibiting NMDAR excitotoxicity and the production of nitric oxide without affecting NMDAR function (Sattler et al., 1999; Ugalde-Triviño and Díaz-Guerra, 2021). Consequently, NA1 has potential as an effective neuroprotective agent in ischemic stroke (Aarts et al., 2002; Xu et al., 2024). After intravenous injection, NA1 can traverse the BBB and inhibit signaling pathways that lead to neuronal excitotoxicity. NA1 has been shown to reduce stroke-induced damage in preclinical studies involving mouse, rat, and primate models, particularly when reperfusion is achieved rapidly (Sun et al., 2008; Bråtane et al., 2011; Cook et al., 2012). A clinical study found that NA1 did not significantly improve clinical outcomes for the entire study population undergoing endovascular thrombectomy for acute ischemic stroke. However, improved outcomes were observed in patients who did not concurrently receive alteplase, suggesting that alteplase may influence the efficacy of NA1 and indicating that these two treatments may potentially interact (Hill et al., 2020).

### Neuroinflammation

Inflammation plays a critical role in the pathology of ischemic stroke and is present throughout nearly all stages of the condition. Neuroinflammation typically begins with the release of damage-associated molecular patterns from damaged or dead cells (**Figure 2B**). Damage-associated molecular patterns such as adenosine, heat shock proteins, and interleukin (IL)-33 are recognized by immune cells, leading to the activation of a range of downstream signaling pathways (Gadani et al., 2015; Shichita et al., 2017; Zhu et al., 2022). During the inflammatory response, immune cells, including microglia, macrophages, and T lymphocytes, are activated (Gelderblom et al., 2009; Iadecola and Anrather, 2011; Liang et al., 2023), which stimulates the secretion of inflammation-related cytokines and chemokines such as monocyte chemoattractant protein-1 (Huang et al., 2006; Mechtouff et al., 2020). Elevated levels of adhesion molecules facilitate the adherence of white blood cells to vascular surfaces, thus promoting immune cell infiltration into the affected area (Zhang et al., 1998; Xie et al., 2022).

Elevated levels of pro-inflammatory cytokines result in the activation of endothelial cells and pericytes, leading to BBB disruption (Stanimirovic and Satoh, 2000; Liebner et al., 2018) accompanied by the release of proteins such as von Willebrand factor and nerve growth factor (Ishitsuka et al., 2012; Gragnano et al., 2017). BBB leakage leads to cerebral edema and an increase in the level of astrocytic aquaporin 4 (Badaut et al., 2002, 2007).

Factors such as monocyte chemoattractant protein-1, von Willebrand factor, nerve growth factor, and aquaporin 4 promote the adhesion of immune cells to the vascular wall and facilitate their infiltration into the central nervous system (CNS), thereby contributing to BBB breakdown and cellular edema. This cascade initiates an irreversible vicious cycle in which the initial inflammation damages the BBB, allowing peripheral neutrophils to migrate into the injured brain; these infiltrating neutrophils then release pro-inflammatory cytokines, produce ROS, and activate MMPs, further disrupting the BBB and perpetuating the inflammatory response.

Rapamycin, an inhibitor of mammalian target of rapamycin (mTOR), exhibits potential therapeutic effects in ischemic stroke through a variety of mechanisms. Notably, it promotes cellular autophagy, which aids in clearing damaged organelles and proteins, thereby reducing cellular damage (Tsou et al., 2022). Additionally, rapamycin suppresses immune responses by inhibiting IL-2, which can further reduce inflammatory reactions. It also promotes the recovery of endothelial function and provides neuroprotection by influencing the functions of neurovascular unit cells, including neurons, astrocytes, and microglia (Hadley et al., 2018).

Curcumin (CUR), a polyphenolic compound derived from *Curcuma longa*, has also been shown to offer protective effects against ischemic stroke. Studies indicate that CUR can suppress the inflammatory response associated with cerebral ischemia-reperfusion injury. It achieves this by reducing tissue concentrations of pro-inflammatory cytokines such as IL-6 and tumor necrosis factor-alpha (TNF-α), downregulating the expression of nuclear factor kappa-light-chain-enhancer of activated B cells (NF-κB), and inhibiting that of cyclooxygenase-2, cell adhesion molecule-1, and MMP-9 (Ran et al., 2021; Subedi and Gaire, 2021). Elevated MMP-9 levels are highly correlated with BBB damage, increased infarct volume, and greater stroke severity (Subedi and Gaire, 2021). A recent study demonstrated that in an animal model of middle cerebral artery occlusion/reperfusion (MCAO/R), pretreatment with CUR contributed to the preservation of BBB integrity and synaptic remodeling in hippocampal neurons (Wu et al., 2021).

Pioglitazone (PGZ), a drug commonly used for the treatment of type 2 diabetes, improves insulin sensitivity through the activation of peroxisome proliferator-activated receptor gamma (PPARG). This receptor also has anti-inflammatory properties, suppressing the activation of NF-κB and reducing the nucleotide oligomerization-like receptor protein 3 (NLRP3) inflammasome-dependent production of IL-1β and IL-18 (Yang et al., 2021). These properties of PGZ highlight its potential to serve as a neuroprotective agent. Furthermore, a clinical trial showed that taking PGZ can significantly reduce the risk of recurrence in patients at high risk of recurrence after an ischemic stroke or transient ischemic attack (Kernan et al., 2017). Notably, however, PGZ can also increase the risk of fractures.

Fingolimod (FTY720), a sphingosine-1-phosphate receptor modulator, functions by preventing lymphocyte migration from lymphoid organs, thereby reducing inflammation and mitigating autoimmune responses. Recent studies have demonstrated that FTY720 can ameliorate ischemia-related neurodegeneration, although the precise underlying mechanism remains unclear. In models of transient MCAO (tMCAO), FTY720 reportedly decreased infarct size and improved ischemic stroke outcomes by limiting lymphocyte-mediated thrombotic interactions in the brain vasculature, rather than providing direct neuronal protection (Kraft et al., 2013; Naseh et al., 2021). FTY720 was found to reduce inflammation following ischemic brain injury by modulating the HMGB1/TLR4/NF-κB signaling pathway (Xing et al., 2024). Notably, the ameliorative effects of FTY720 on ischemic brain injury may de dose-dependent.

### Hypoxia and oxidative stress

The formation of blood clots during ischemic stroke can reduce oxygen supply to brain tissue (Denecke et al., 2022). In response to low oxygen levels, intracellular oxygen sensors such as hypoxia-inducible factor-1 alpha (HIF-1α), endoplasmic reticulum oxidoreductin 1-alpha (ERO1-α), and heme-regulated inhibitor (HRI) are activated (**Figure 2C**). In the oxygen-glucose deprivation (OGD) cell model, the expression of all three molecules is increased, leading to hypoxia and heightened expression of genes associated with oxygen deprivation (May et al., 2005). ROS production induces oxidative stress, which significantly damages lipids, proteins, and DNA, and ultimately results in mitochondrial damage (Zhang et al., 2019; Maida et al., 2020). This cascade then triggers the production of even more ROS. Additionally, the activities of antioxidant enzymes are decreased during ischemic stroke, which further exacerbates ROS-induced damage (Huang et al., 2022).

Glutathione (GSH) plays a crucial role in protecting cellular components from damage caused by ROS, free radicals, peroxides, and heavy metals (Pompella et al., 2003). It is a key reducing agent within the antioxidant system and is more abundant than other reducing agents such as cysteine and selenocysteine. Additionally, GSH serves as the primary cellular antioxidant for maintaining redox balance (Martínez-Revelles et al., 2016). However, GSH levels significantly decrease following ischemic stroke, which diminishes the activity of glutathione peroxidase 4 (GPX4), thereby disrupting the metabolism of lipid peroxides typically mediated by this enzyme. This leads to Fe^2+^-catalyzed lipid oxidation via the Fenton reaction, producing ROS and promoting ferroptosis, an iron-dependent form of programmed cell death characterized by the accumulation of lipid peroxides. Ferroptosis is frequently observed following ischemic stroke (Dixon et al., 2012).

Edaravone (EDA) is an antioxidant used to eliminate free radicals in the treatment of ischemic stroke and has been approved for marketing in Japan (Miyaji et al., 2015). Preclinical and clinical studies have demonstrated that EDA effectively prevents cerebral edema caused by ischemic stroke and reperfusion injury (Yoshida et al., 2006; Xu et al., 2021; Teng et al., 2024).

Simvastatin is a 3-hydroxy-3-methylglutarylcoenzyme A (HMG-CoA) reductase inhibitor and a commonly used oral lipid-lowering drug. Clinical studies have reported that simvastatin significantly reduces the incidence of ischemic stroke, regardless of serum cholesterol concentrations (Sacks et al., 1996; Blauw et al., 1997; Montaner et al., 2016). This suggests that simvastatin offers neuroprotective benefits beyond its established role in lowering the levels of cholesterol levels (Vaughan and Delanty, 1999). Animal studies indicate that simvastatin may protect against ischemic stroke through its influence on different isoforms of nitric oxide synthase (NOS), potentially restoring cerebral blood flow (Cimino et al., 2005; Sironi et al., 2006). Additionally, simvastatin can reduce vascular inflammation (Pruefer et al., 1999), modulate cytokine production, and promote angiogenesis (Kureishi et al., 2000). However, the neuroprotective effects of simvastatin in ischemic stroke remain controversial (Duval, 2000).

Melatonin possesses antioxidant and stress-relieving properties and is capable of neutralizing ROS and RNS, including hydrogen peroxide, singlet oxygen, hydroxyl radicals, peroxynitrite anions, and nitric oxide (Dun-xian et al., 2002). In addition to directly eliminating these harmful species, melatonin enhances the activity of antioxidant enzymes and inhibits that of pro-oxidant ones, thereby reducing oxidative stress. Research has demonstrated that melatonin improves mitochondrial resistance to oxidative stress by counteracting the toxic effects of metal ions (Reiter et al., 2016). Notably, as a lipid-soluble free radical scavenger, melatonin can cross the BBB and exert protective effects in the CNS.

## Nanoparticles in Ischemic Stroke Treatment

**Figure 3:**
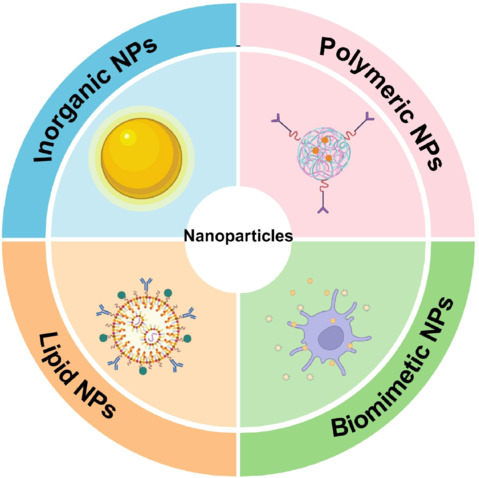
Summary of NPs as drug delivery systems in ischemic stroke. The common NPs for ischemic stroke include inorganic NPs, polymeric NPs, lipid NPs, and biomimetic NPs. Created with BioRender.com. NPs: Nanoparticles.

To enhance the efficacy and safety of the drugs discussed in the first section, a variety of drug delivery systems have been developed. NP-based drug delivery systems play a significant role in the treatment of ischemic stroke and can be broadly categorized into four types—inorganic, polymeric, lipidic, and biomimetic (**Figure 3**).

### Inorganic nanoparticles

Studies have indicated that inorganic NPs can significantly contribute to the treatment of ischemic stroke (**Table 1**), benefiting from their excellent antioxidant properties, which are similar to those of carbon-based and metal oxide NPs (Fluri et al., 2015; Ge et al., 2022).

**Table 1:** Inorganic NPs in the treatment of ischemic stroke

NPs	Design	Synthesis methods	Therapeutic mechanisms	References
Fullerene NPs	C_60_(OH)_18–22_	Undescribed	Reducing oxidative damage and aquaporin-1 expression	Darabi and Mohammadi, 2017
CeO_2_ NPs	CeO_2_, TPP, ROF, DSPE-PEG	High-temperature method	Reducing mitochondrial ROS and improving antioxidant enzyme activities	Liao et al., 2024
	CeO_2_, NBP, PEG	High-temperature method	Scavenging ROS and repairing BBB damage	Li et al., 2022b; Liu et al., 2023
	Ultrathin CeO_2_ with surface strain	Driving surface intrinsic strain through thickness cutting	Significantly enhancing the SOD-mimetic activity and reducing toxicity	Liu et al., 2023
Cu_2_O NPs	Cu_4.6_O, zein-Se-Se-DHA, rt-PA, platelet cell membrane	Reducing CuCl_2_ with ascorbic acid	Scavenging ROS and promoting M2 polarization	Kong et al., 2024
Mn_3_O_4_ NPs	Mn_3_O_4_, HSA	Solvothermal method	Mitigating oxidative stress and endoplasmic reticulum stress	Huang et al., 2022
	Mn(III) complex	Reducing Mn(OAc)_2_ by diamines	Increasing oxygen production and decreasing HO·	Ning et al., 2020
POM nanoclusters	(NH_4_)_6_Mo_7_O_24_·4H_2_O	Stirring in ultrapure water and reducing Mo^6+^ to Mo^5+^ by ascorbic acid	Scavenging ROS and decreasing quantities of pro-inflammatory cytokines	Li et al., 2019

BBB: Blood–brain barrier; DHA: docosahexaenoic acid; DSPE: 1,2-distearoyl-sn-glycero-3-phosphoethanolamine; GSH: glutathione; HSA: human serum albumin; NBP: DL-3-n-butylphthalide; PEG: polyethylene glycol; POM: polyoxometalates; ROF: roflumilast; ROS: reactive oxygen species; rt-PA: recombinant tissue-type plasminogen activator; SOD: superoxide dismutase; TPP: triphenylphosphine.

Carbon-based NPs, particularly fullerenes and their derivatives, are known for their ability to eliminate free radicals and exceptional neuroprotective properties (Fernandes et al., 2018). Vani et al. (2016) demonstrated that, in a rat model of ischemic stroke, fullerene NPs markedly enhance the treatment of cerebral infarction alongside reducing oxidative and nitrosative stress. Specifically, treatment with fullerenes resulted in decreased levels of malondialdehyde (MDA) and nitrates in the ischemic hemispheres. Additionally, fullerene NPs increased GSH content and superoxide dismutase (SOD) activity in ischemic brains, leading to significant improvements in neurological deficits compared to that observed with the control treatment (Vani et al., 2016).

**Figure 4:**
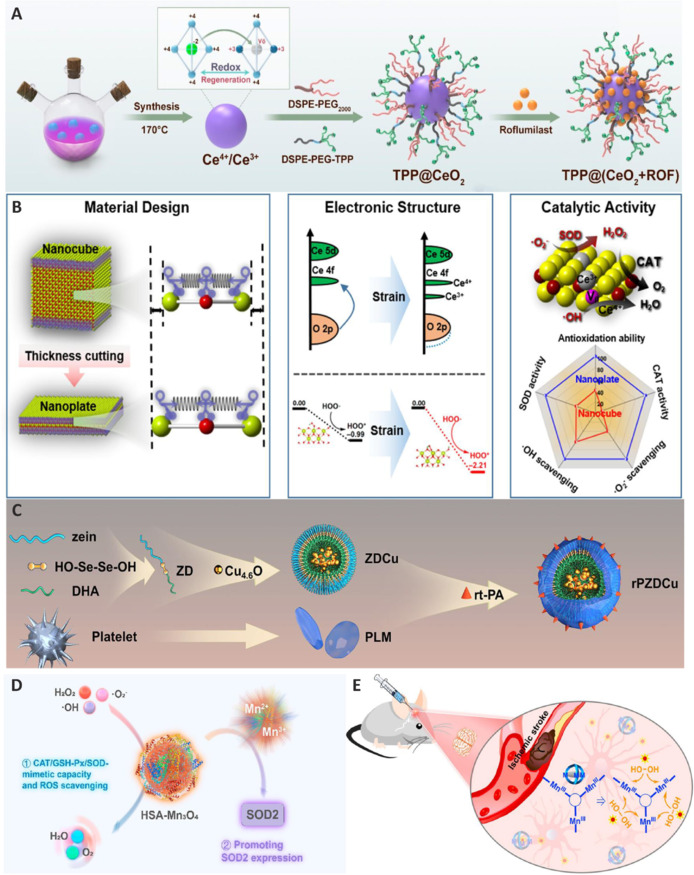
Inorganic NPs for treating ischemic stroke. (A) Fabrication of TPP@(CeO_2_+ROF). Reproduced with permission from Liao et al. (2024). Copyright © 2024 American Chemical Society. (B) Surface strain in ultrathin ceria nanoplates enhances SOD-mimetic activity. Reproduced with permission from Liu et al. (2023). Copyright © 2023 American Chemical Society. (C) Fabrication of rPZDCu. Reproduced with permission from Kong et al. (2024). Copyright © 2024 American Chemical Society. (D) HSA-Mn_3_O_4_ exhibits multifunctionality: CAT, GSH-Px, SOD mimetic activity, ROS scavenging, and promotion of SOD2 expression. Reproduced with permission from Huang et al. (2022). Copyright © 2021 American Chemical Society. (E) Salen-based tri-manganese metallocryptands significantly enhance oxygen production while reducing hydroxyl radical (·OH) formation. Reproduced with permission from Ning et al. (2020). Copyright © 2020 American Chemical Society. CAT: Catalase; DHA: docosahexaenoic acid; DSPE: 1,2-distearoyl-sn-glycero-3-phosphoethanolamine; GSH: glutathione; GSH-Px: glutathione peroxidase; HSA: human serum albumin; NPs: nanoparticles; PEG: polyethylene glycol; PLM: platelet cell membrane; PLM: platelet cell membrane; ROF: roflumilast; ROS: reactive oxygen species; rt-PA: recombinant human tissue plasminogen activator; SOD: superoxide dismutase; TPP: triphenylphosphine.

Compared to metal NPs, metal oxide NPs exhibit greater oxidation resistance in CNS diseases (Anu Mary Ealia and Saravanakumar, 2017; Faisal and Kumar, 2017). Among the latter, cerium oxide NPs (CeO_2_ NPs) have garnered considerable attention owing to their potent antioxidant properties, which arise from their unique ability to form oxygen vacancies as well as exhibit low-valence states. These characteristics make CeO_2_ NPs particularly effective at alleviating oxidative stress, a significant factor in the pathophysiology of ischemic stroke (Estevez et al., 2011). Liao et al. (2024) developed a mitochondria-targeted nanosystem that combines triphenylphosphine (TPP) modifications, CeO_2_, and roflumilast (ROF) [TPP@(CeO_2_+ROF)] for the treatment of ischemic stroke (**Figure 4A**). The positive charge of TPP enables selective accumulation in negatively charged mitochondria, allowing for targeted drug delivery to mitochondria in the brain. The authors reported that TPP@(CeO_2_+ROF) effectively targeted mitochondria, reduced mitochondrial ROS, restored mitochondrial membrane potential, and improved the activities of antioxidant enzymes such as glutathione peroxidase (GPx) and SOD. In a rat model of MCAO, TPP@(CeO_2_+ROF) demonstrated strong brain-targeting capabilities, traversed the BBB, reduced cerebral infarct volume, decreased brain edema, and improved neurological function. Histological analysis through hematoxylin and eosin staining and cerium biodistribution studies further indicated that the NPs displayed excellent long-term biosafety. Furthermore, TPP@(CeO_2_+ROF) exhibited a notable ability to reverse BBB damage caused by ischemic stroke (Liao et al., 2024). Dl-3-n-butylphthalide (NBP), a new FDA-approved drug developed in China, has been shown to improve CNS damage and promote functional recovery in patients following acute ischemic stroke (Wang et al., 2018). To combine the neurovascular repair properties of NBP with the ROS scavenging capabilities of CeO_2_ for the treatment of ischemic stroke, Li et al. (2022) synthesized ultra-small CeO_2_ NPs as carriers of NBP (NBP-CeO_2_ NPs) employing a high-temperature method. These NPs exhibited significant ROS scavenging capabilities in both hippocampal neurons (HT22) and brain microvascular endothelial cells subjected to oxygen-glucose deprivation/reoxygenation (OGD/R). *In vitro* experiments further demonstrated that approximately 79% of brain microvascular endothelial cells maintained a relatively healthy mitochondrial network morphology. The NPs preserved mitochondrial morphology, membrane potential, and function, which were significantly disrupted following OGD/R, thereby inhibiting apoptosis. In a mouse model of MCAO/R, NBP-CeO_2_ NPs protected the integrity of the BBB, resulting in reduced cerebral infarction and edema. MCAO/R model animals treated with NBP-CeO_2_ NPs exhibited an average reduction of approximately 10.6% in the size of the cerebral infarct relative to untreated controls. Additionally, NBP-CeO_2_ NP treatment inhibited neuroinflammation and neuronal apoptosis, contributing to improved spatial learning and sensorimotor function in long-term neurobehavioral tests. A cytotoxicity study indicated that NBP-CeO_2_ NPs did not significantly impact the viability of HT22 cells. Moreover, histological findings (H&E staining) revealed that NBP-CeO_2_ NPs had good biocompatibility with the examined organs (Li et al., 2022a).

Despite the significant advantages of metal oxide nanoenzymes, several issues warrant consideration. For instance, the enzyme-mimetic activities of these nanoenzymes are typically 1 to 2 times lower than those of enzymes derived from natural sources (Huang et al., 2019). Additionally, metal oxide nanoenzymes may exhibit dose-dependent toxicity (Ghorbani et al., 2019). An effective strategy for regulating the activity of metal oxide nanoenzymes is to introduce inherent strain by slicing two-dimensional geometric architectures at the atomic scale (Wang et al., 2019). Liu et al. (2023) focused on enhancing the catalytic performance of metal oxide nanozymes, specifically CeO_2_, to mimic the activity of antioxidant enzymes for the treatment of ischemic stroke (**Figure 4B**). They engineered surface strain in ultrathin ceria nanoplates, achieving approximately 3% in-plane and 10% out-of-plane tensile strain. This strain was validated through density functional theory calculations and experimental synthesis. Compared with nanocubes, ultrathin nanoplates exhibiting surface strain displayed increased Ce–O bond covalency, which significantly enhanced SOD-mimetic activity by approximately 2.6-fold and the total antioxidant effect by 2.5-fold. Overall, these nanoplates demonstrated significant therapeutic efficacy both *in vitro* and *in vivo* (Liu et al., 2023).

Ultrasmall copper (Cu) and cuprous oxide (Cu_2_O) NPs are recognized for their ROS-scavenging catalytic activity (Gawande et al., 2016). Kong et al. (2024) introduced a novel multifunctional NP (rPZDCu) composed of recombinant human tissue plasminogen activator (rt-PA), platelet cell membrane (PLM), and ZDCu (Cu4.6O@zein-Se-Se-DHA), for the treatment of ischemic stroke. This NP exhibited thrombolytic, ROS scavenging, and neuroprotective properties (**Figure 4C**). *In vitro* studies demonstrated that rPZDCu has significant ROS scavenging capabilities, particularly in neutralizing superoxide (O_2_^–^), hydroxyl radicals (·OH), and hydrogen peroxide (H_2_O_2_), achieving over 80% scavenging efficiency at a concentration of 500 μg/mL. Furthermore, rPZDCu treatment decreased the levels of pro-inflammatory cytokines, including IL-1β, IL-6, and TNF-α, and promoted M2 polarization, which is associated with anti-inflammatory processes and tissue repair. In vivo studies using MCAO models showed that rPZDCu improved thrombus targeting and thrombolytic performance, while also enhancing neuroprotection and recovery of neurological and behavioral functions. Additionally, histological assessment of major organs from rats revealed that rPZDCu caused no detectable damage to the heart, liver, spleen, lungs, or kidneys. Serum biochemical tests conducted to assess the potential toxicity of rPZDCu did not identify abnormalities in key indicators of liver and kidney function (Kong et al., 2024).

Manganese oxide (Mn_3_O_4_) is useful for ischemic stroke treatment because of its unique catalytic properties and neuroprotective potential. Mn_3_O_4_ NPs exhibit intrinsic antioxidant activity, effectively mimicking the functions of antioxidant enzymes such as SOD and catalase (Li et al., 2021a). Huang et al. (2022) synthesized a biomimetic human serum albumin (HSA)-Mn_3_O_4_ nanozyme to mitigate oxidative and endoplasmic reticulum stress in nerve cells following ischemic stroke (**Figure 4D**). HSA-Mn_3_O_4_ demonstrated strong ROS scavenging ability and long circulation time, which collectively contributed to its therapeutic efficacy in treating ischemic stroke. In OGD model cells, the neuroprotective effects of this nanozyme were attributed to its ability to reduce apoptosis and maintain mitochondrial function. Additionally, HSA-Mn_3_O_4_ exposure resulted in significantly reduced hemolysis compared to Mn_3_O_4_ treatment alone, demonstrating that modification with HSA can enhance biocompatibility. It has been reported that functionalizing Mn_3_O_4_ NPs with organic ligands enhances their therapeutic potential (Huang et al., 2022). Ning et al. (2020) fabricated a series of salen-based tri-manganese metallocryptands that can mimic the activity of the enzyme catalase, resulting in decreased production of HO· and enhanced neuroprotection (**Figure 4E**). Complex 1, which displayed the greatest potential, exhibited significantly enhanced catalase-like activity compared with that observed with monomeric Mn(salen), resulting in a 6.8-fold increase in oxygen production and a 50-fold reduction in HO· formation. Rats subjected to cerebral ischemia and treated with Complex 1 showed a marked reduction in brain infarct volumes and better neurological outcomes compared to rats in the control groups (Ning et al., 2020).

Recent studies have shown that ultrasmall molybdenum (Mo)-based polyoxometalate (POM) nanoclusters can effectively scavenge ROS by altering their redox status (Ni et al., 2018; Li et al., 2023b; Yan et al., 2023b). Additionally, POMs demonstrate notable efficacy in the treatment of CNS diseases (Gao et al., 2014). Li et al. (2019) investigated the potential of POM nanoclusters as nano-antioxidants for protecting neurons from oxidative damage in cerebral ischemia/reperfusion injury. Intrathecal injection allowed for the precise targeting of POM nanoclusters to the ischemic zone. The results demonstrated that the POM nanoclusters delivered in this manner were effective at scavenging ROS, reducing infarct size, and lowering the concentrations of pro-inflammatory cytokines, including TNF-α and IL-6. Furthermore, toxicity evaluation revealed no adverse effects on major organs and only minimal neurotoxicity. Importantly, neurological function was significantly improved in rat models.

### Polymeric nanoparticles

**Figure 5:**
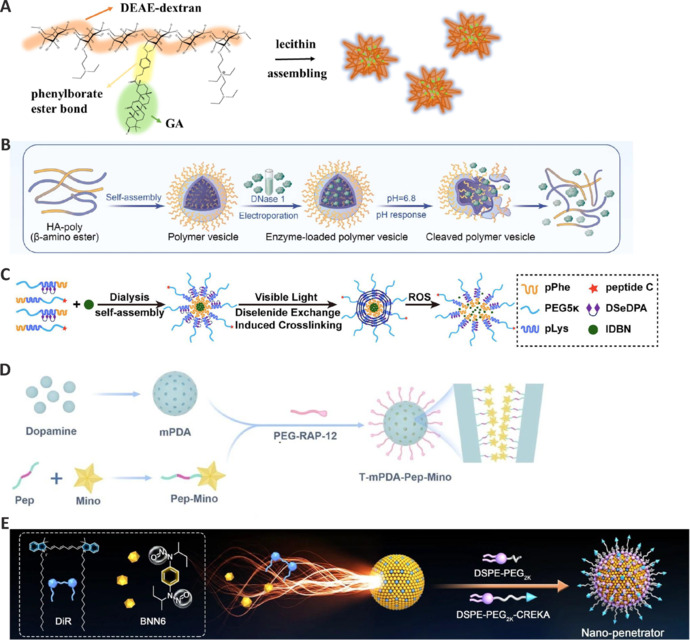
Poly NPs for treating ischemic stroke. (A) The fabrication of DGA. Reproduced with permission from Jin et al. (2023). Copyright © 2022 Elsevier B.V. (B) Self-assembled poly NPs released DNase 1 in response to the acidic microenvironment of the ischemic brain. Reproduced with permission from Li et al. (2024). Copyright © 2023 Elsevier Ltd. (C) mPLSeP modified with CREKA peptide delivered IDBN. Reproduced with permission from Li et al. (2023). Copyright © 2023 American Chemical Society. (D) Fabrication of T-mPDA-Pep-Mino. Reproduced with permission from Wu et al. (2023). Copyright © 2023 Springer Nature Limited. (E) DiR-BNN6 fuel pair modified by PEGylation. Reproduced with permission from Zhang et al. (2023). Copyright © 2023 Springer Nature Limited. BNN6: N,N′-di-sec-butyl-N, N′-dinitroso-1,4-phenylenediamine; CREAK: Cys-Arg-Glu-Lys-Ala; DEAE: diethylaminoethylen; DiR: 1,1′-dioctadecyl-3,3,3′,3′-tetramethylindotricarbocyanine iodide; DseDPA: 3,3′-diselanediyldipropionic acid; DSPE: 1,2-distearoyl-sn-glycero-3-phosphoethanolamine; GA: glycyrrhetinic acid; HA: hyaluronic acid; IDBN: idebenone; Mino: 9-amino minocycline; mPDA: mesoporous polydopamine; NPs: nanoparticles; PEG: polyethylene glycol; Pep: MMP-2 responsive peptide; pLys: phosphorylated lysine; pPhe: phosphorylated phenylalanine; RAP-12: receptor-associated protein; ROS: reactive oxygen species.

Polymeric NPs have emerged as promising delivery systems in the treatment of ischemic stroke (**Table 2**), offering unique platforms for the targeted delivery of therapeutic agents and allowing for enhanced efficacy (Zhu et al., 2024). Diethylaminoethylene (DEAE)-dextran has shown potential as an effective hydrophilic backbone (Siewert et al., 2019). Jin et al. (2023) synthesized glycyrrhetinic acid (GA)-conjugated DEAE-dextran (DGA) NPs incorporating phenyl boronate ester linkers for ROS responsiveness (**Figure 5A**), an approach that enhanced GA solubility and therapeutic efficacy. The NPs effectively inhibited high mobility group box 1 (HMGB1) and induced a switch in microglia from an M1 to an M2 phenotype (Jin et al., 2023).

**Table 2:** Poly NPs in ischemic stroke treatment

Loaded drugs	Targeting ligands/methods	Polymers	Therapeutic mechanisms	References
Glycyrrhetinic acid	Phenyl boronate ester linkers	DEAE-dextran	Inhibiting HMGB-1 and switching M1 microglia to M2 microglia	Jin et al., 2023
DNase 1	Poly (β-amino ester)	Hyaluronic acid-poly (β-amino ester)	Inhibiting NETs, preventing platelet from activating NETs through the TLR4 pathway and downregulating HMGB-1	Li et al., 2024
IDBN	CREKA peptide and diselenide cross-linkers	mPEG_5K_-pLys_12_(Se)-pPhe_20_-CH_3_	Inhibiting neuronal ferroptosis and glial overactivation	Li et al., 2023a
Polydopamine	MMP-2 responsive peptide and brain-targeting peptide	Polydopamine, PEG	Scavenging ROS and regulating microglial polarization	Wu et al., 2023
Piceatannol	PLGA with aspect ratio (AR) of 5, which easily taken up by neutrophils	PLGA	Inhibiting p-Syk signaling in neutrophils and microglia to repair BBB	Song et al., 2023
RAPA	ROS-sensitive boronic ester and erythrocyte membrane containing a stroke-specific peptide	Sulfated chitosan	Promoting the shift of microglia from M1 phenotype to M2 phenotype	Cao et al., 2024
Edaravone	mPEG−P(Glu-coCys^2^)	mPEG−P(Glu-coCys^2^)	Inhibiting ferroptosis and scavenging ROS	Zhang et al., 2024b
BNN6	CREKA peptide	DSPE-PEG_2K_ and DEPE-PEG_2K_-CREKA	Continuously providing NO to reduce thrombus	Zhang et al., 2023

CREKA: Cysteine-arginine-glutamicacid-lysine-alanine; DEAE: diethylaminoethyl; HMGB1: high mobility group box 1; IDBN: idebenone; MMP-2: matrix metalloproteinase-2; NETs: neutrophil extracellular traps; NO: nitric oxide; NPs: nanoparticles; PEG: polyethylene glycol; PLGA: poly(lactic-co-glycolic acid); p-Syk: phosphorylated spleen tyrosine kinase; ROS: TLR4: toll-like receptor 4.

Thrombo-inflammation is a major contributor to ischemia-reperfusion injury. It occurs when neutrophils produce neutrophil extracellular traps (NETs), which then interact with platelets and neutrophils, thereby accelerating infarct progression. Deoxyribonuclease I (DNase 1) is the main enzyme responsible for NET degradation. However, it exhibits several limitations, namely, rapid deactivation and low efficiency in targeting the ischemic brain (Chen et al., 2022; Li et al., 2022a). To overcome these challenges, Li et al. (2024) developed a pH-responsive polymersome for enhancing the delivery of DNase 1 to the brain. The polymersomes, formed by self-assembling hyaluronic acid-poly(β-amino ester) (HB), were loaded with DNase 1 through electroporation. In an acidic environment, HB undergoes protonation and rapidly decomposes, enabling the NPs to release DNase 1 in response to the acidic microenvironment of the ischemic brain, thereby facilitating NET degradation (**Figure 5B**). This process disrupts the thrombus structure and inhibits microthrombosis. DNase 1 also reduces histone stability in NETs, which prevents platelet activation through the TLR4 pathway, and subsequently downregulates HMGB-1, suppressing further NET formation (Li et al., 2024).

Polyethylene glycol-grafted poly-L-lysine (PEG-PLL) is a synthetic polymer commonly used in drug delivery systems and tissue engineering. It enhances the solubility and stability of drugs, exhibits good biocompatibility and low toxicity, and its surface can be further modified for targeted therapy. Based on this, Li et al. (2023) developed CREKA peptide-modified micelles with diselenide cross-linkers and the amphiphilic triblock polymer, mPEG5K-pLys12(Se)-pPhe20-CH3 (mPLSeP), for targeted treatment of cerebral ischemia-reperfusion injury. These micelles can bind to microthrombi in ischemic areas and release the drug in a ROS-dependent manner. These micelles were used to deliver idebenone, an antioxidant, to the ischemic brain, where it inhibited neuronal ferroptosis and glial overactivation (**Figure 5C**). In a rat model, the micelles enhanced drug accumulation in the brain, reduced infarct volume, and improved neurological function, underscoring their potential as a pleiotropic system for stroke treatment (Li et al., 2023a).

Polydopamine (PDA) is a melanin-like substance with multiple properties, including antioxidant, photothermal conversion, biocompatibility, and biodegradation effects (Liu et al., 2014). Wu et al. (2023) designed an MMP-2-responsive brain-targeting nanosystem, named T-mPDA-Pep-Mino, using mesoporous PDA NPs that were functionalized with minocycline-conjugated MMP-2-responsive peptides and PEGylated brain-targeting peptides (**Figure 5D**). This strategy was used as MMP-2 activity is specifically elevated after infarction, while minocycline acts as a selective inhibitor of microglial pro-inflammatory polarization, an effect that is achieved through the modulation of the NF-κB signaling pathway. This nanosystem reduced infarct volume and significantly improved neurological recovery both *in vitro* and *in vivo*, suggesting that a pathogenesis-tailored strategy guided by this nanosystem may hold promise for addressing the challenges of reperfusion-induced neuroinflammation (Wu et al., 2023). PLGA is a biodegradable polymer formed through the random copolymerization of lactic acid and glycolic acid. Besides its excellent biocompatibility, PLGA also possesses good encapsulation and film-forming properties, making it widely used in pharmaceuticals, engineering materials for health care, and advanced industrial fields (Martins et al., 2018). Song et al. (2023) developed rod-shaped PLGA NPs with an aspect ratio (AR) of 5 (AR5) and loaded them with piceatannol (Pic). These NPs (Pic@AR5) can effectively target and hinder the interaction between neutrophils and inflamed endothelial cells, thus blocking neutrophil infiltration into the BBB. Pic@AR5 NPs could be efficiently taken up by neutrophils *in vitro* and alleviated neuroinflammation in ischemic stroke model mice.

Sulfated chitosan exhibits excellent anticoagulant activity and outstanding biocompatibility (Yu et al., 2021). Cao et al. (2024) developed a RAPA@tRPCS nanocarrier, which was based on a sulfated chitosan polymer core, modified with a ROS-sensitive boronic ester and an erythrocyte membrane containing a stroke-targeting peptide. The RAPA@tRPCS showed a good ability to target ischemic sites and controlled rapamycin (RAPA) release behavior in response to ROS, thereby facilitating significant drug accumulation in damaged brain tissues. Additionally, RAPA@tRPCS treatment promoted an M1 to an M2 phenotypic shift in microglia, reduced inflammation, and promoted tissue repair. This nanocarrier also ameliorated angiogenesis and neurogenesis, leading to improved stroke recovery (Cao et al., 2024).

Zhang et al. (2024) synthesized a poly(amino acid) nanogel loaded with EDA (NG/EDA) composed of methoxy poly(ethylene glycol) and poly(L-glutamic acid-co-L-cystine). NG/EDA is a dual-responsive system that selectively releases EDA in acidic environments, followed by an EDA-induced increase in the levels of GSH. NG/EDA displayed a regular sub-spherical morphology with an average hydrodynamic diameter of approximately 112.3 nm. Moreover, NG/EDA effectively accumulated at the ischemic injury site in pMCAO mice, indicating that it readily crossed the BBB. Furthermore, NG/EDA inhibited ferroptosis, a type of cell death related to ischemic injury, accompanied by reductions in Fe²⁺, ROS, and MDA levels in neurons, relative to that seen with free EDA. The levels of GSH were also higher in neurons treated with NG/EDA, indicative of enhanced antioxidant activity.

NO can dilate blood vessels, consequently increasing blood supply and reducing blood pressure. It can protect against tissue damage due to low blood supply, thus showing promising prospects for ischemic stroke treatment (Wan et al., 2019). Zhang et al. (2023) presented a self-fueled, nano-penetrator nanoassembly (T-BD NA) containing 1,1ʹ-dioctadecyl-3,3,3ʹ,3ʹ-tetramethylindotricarbocyanine iodide (DiR) and an NO donor (N,Nʹ-di-sec-butyl-N,Nʹ-dinitroso-1,4-phenylenediamine) (BNN6) and PEGylated for the nonpharmaceutical interventions of thrombosis and ischemic stroke (**Figure 5E**). The nano-penetrator was optimized at a 1:3 DiR: BNN6 molar ratio for efficient NO generation. Besides, fibrin-homing decoration was used to optimize the targeting behavior of the nano-penetrator. The authors reported that the nano-penetrator achieved high DiR and BNN6 loading rates (41.1% and 33.9%, respectively), implying that 75% of the components in the nano-penetrator serve as fuels, thereby facilitating efficient NO generation. Notably, the nano-penetrator induced a marked reduction in thrombus volume and an increase in fibrin and hemoglobin expression in supernatants, as evidenced in *in vitro* thrombolysis assays. Moreover, none of the formulations induced hemolysis or exerted potential cytotoxicity, either with or without laser irradiation, indicating the excellent biocompatibility of T-BD NAs for intravenous injection applications. In summary, this system exhibited superior efficacy and safety compared to conventional thrombolytic drugs, highlighting its promising potential as an alternative for clinical applications in the treatment of thrombotic cerebro-cardiovascular diseases (Zhang et al., 2023).

### Lipid nanoparticles

Lipid nanoparticles (LNPs) are prepared using biodegradable and biocompatible amphiphiles such as fatty acids and phospholipids (Yaghmur and Glatter, 2009). The amphiphilic properties of these components enable LNPs to encapsulate both hydrophobic and hydrophilic substances, offering distinct structural and functional benefits. Among the LNPs, liposomes are the most extensively studied for their potential for drug delivery across the BBB for the treatment of ischemic stroke. Numerous efforts have been devoted to enhancing BBB targeting by modifying the surface chemistry of LNPs (**Table 3**). This includes the use of specific ligands and surfactants, such as Tween 80, which facilitate the adsorption of plasma proteins that mimic low-density lipoproteins, thereby improving NP internalization by endothelial cells (Kreuter et al., 2002).

**Table 3:** LNPs in ischemic stroke treatment

Loaded drugs	Targeting ligands/methods	Synthesis methods	Therapeutic mechanisms	References
Cl-amidine	ROS responsive ketothiol and CAEKA peptide	Double emulsion-solvent evaporation method	Inhibiting PAD4 and cGAS-STING pathway	Sun et al., 2023
tPA and aspirin	Ada-Se-lipo	Ester−amide reaction	Inhibiting platelet aggregation, reducing the infiltration of neutrophils, and mitigating tPA-induced inflammation	Quan et al., 2023
Paeonol and polymetformin	Platelet membranes	Conventional thin-film hydration method	Scavenging ROS and reprogramming microglial from M1 phenotype to M2 phenotype	Tang et al., 2024
9-Phenanthrol	T7 (HAIYPRH)	Ethanol injection method	Inhibiting TRPM4 channel	Liu et al., 2024
Protoporphyrin IX	Platelet membranes	Bio-orthogonal click reaction	Inducing specific M2 polarization of microglia	Li et al., 2021b
3-n-butylphthalide	B-rLNPs	Solvent diffusion method	Inhibiting ferroptosis and scavenging ROS	Han et al., 2024

B-rLNPs: Brain–derived reconstituted lipid nanoparticles; cGAS-STING: cyclic GMP-AMP synthase-stimulator of interferon genes pathway; LNPs: lipid nanoparticles; PAD4: peptidylargininedeiminase 4; ROS: reactive oxygen species; TRPM4: transient receptor potential melastatin 4.

**Figure 6:**
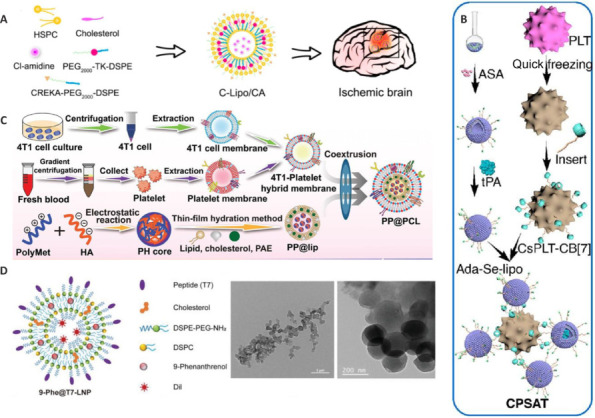
Lipid NPs for treating ischemic stroke. (A) Self-assembly LNPC-Lipo/CA targeted the cerebral ischemia site. Self-assembly of LNPC-Lipo/CA for targeted delivery to cerebral ischemia sites. Reproduced with permission from Sun et al. (2023). Copyright © 2023 American Chemical Society. (B) Fabrication of the CPSAT system. Reproduced with permission from Quan et al. (2023). Copyright © 2023 American Chemical Society. (C) The synthesis of PP@PCL. Reproduced with permission from Tang et al. (2024). Copyright © 2024 Wiley-VCH GmbH. (D) The structure and transmission electron microscopy photos of 9-Phe@T7-LNP. Reproduced with permission from Liu et al. (2024). Copyright © 2024 Wiley-VCH GmbH. Ada: Adamantane; ASA: aspirin; CREAK: Cys-Arg-Glu-Lys-Ala; CsPLT-CB: cryo-shocked platelet-cucurbit[7]uril; Dil: 1,1′-dioctadecyl-3,3,3′,3′-tetramethylindocarbocyanine perchlorate; DSPC: 1,2-distearoyl-sn-glycero-3-phosphocholine; HSPC: hydrogenated soybean phospholipids; Lipo: liposomes; PAE: paeonol; PEG2000-TK-DSPE: polyethylene glycol2000-ketothiol -1,2-distearoyl-sn-glycero-3-phosphoethanolamine; tPA: thrombolysis with tissue plasminogen activator.

Mitochondrial dysfunction in ischemic stroke leads to oxidative stress through increased production of ROS, which then activate downstream inflammatory responses. Alternatively, ROS can function as a trigger for drug release at the site of cerebral ischemia, thereby enabling controlled drug delivery (Huang et al., 2015; Lv et al., 2018). In this regard, ROS-responsive ketothiol (TK) materials are used to construct drug carriers that break and release drugs in response to increased intracellular ROS levels (Pei et al., 2019; Liu and Thayumanavan, 2020; Zhai et al., 2022). Cl-amidine, an inhibitor of peptidylarginine deiminase 4, can suppress NET production from its origin (Hakkim et al., 2010), as well as significantly reduce vascular damage and enhance neovascularization in the ischemic cortex (Kang et al., 2020). Sun et al. (2023) developed a ROS-responsive liposome, CREKA-Lipo/Cl-amidine (C-Lipo/CA), modified with PEG-TK as a ROS-responsive moiety, to enhance ischemic stroke treatment. This system targeted NET generation and inhibited the cyclic guanosine monophosphate (GMP) -adenosine monophosphate (AMP)synthase (cGAS)-stimulator of interferon genes (STING) (cGAS-STING) pathway (**Figure 6A**). The results showed that C-Lipo/CA significantly reduced cerebral infarction and improved neurological outcomes by modulating the immune response and enhancing neuronal survival (Sun et al., 2023). Quan et al. (2023) prepared a mixture of cryo-shocked platelets (CsPLT) and ROS-responsive liposomes (ada-Se-lipo) containing tPA and aspirin (**Figure 6B**). The CsPLT-Ada-Se-lipo (CPSAT) system showed strong attachment to activated platelets, significantly reducing platelet aggregation compared to the control treatment. The system also exhibited potent anti-inflammatory effects in lipopolysaccharide (LPS)-treated RAW264.7 cells, reducing the levels of pro-inflammatory cytokines. In mouse models of mesenteric artery thrombosis and ischemic stroke, the CPSAT system demonstrated notable targeting and therapeutic efficacy, including reducing thrombus size, decreasing infarct volume, and improving blood vessel recanalization (Quan et al., 2023).

It has been reported that 4T1 tumor cell membranes can penetrate the BBB (He et al., 2021) and that platelet membranes can efficiently target ischemic lesions (Kaviarasi et al., 2019; Tang et al., 2023). Leveraging these properties, Tang et al. (2024) coated liposomes loaded with paeonol (PAE) and polymetformin (PolyMet) with a combination of the two membranes, forming PP@PCL (**Figure 6C**). PP@PCL demonstrated excellent BBB penetration and ischemic lesion targeting ability. *In vitro*, PP@PCL exposure led to a significant reduction in neuronal apoptosis and oxidative stress in pheochromocytoma cell line 12 (PC12) cells subjected to OGD/R. Moreover, PP@PCL effectively scavenged ROS, prevented neuroinflammation, and promoted the reprogramming of microglia from an M1 to an M2 phenotype. *In vivo*, PP@PCL reduced infarct volume, facilitated neuronal regeneration, improved neurological function, and enhanced survival rates in rats with induced ischemic stroke (Tang et al., 2024).

9-Phenanthrol (9-Phe), a widely used TRPM4 channel inhibitor, has been confirmed to reduce brain edema (Simard et al., 2012; Wang et al., 2013), and even protect isolated hearts from ischemia-reperfusion injury (Piao et al., 2015). However, 9-Phe displays poor water solubility (Peng et al., 2017) and strong cytotoxicity (Guinamard et al., 2014), which restricts its suitability as a drug candidate. To address these limitations, Liu et al. (2024) constructed a brain-targeted lipid nanoparticle (LNP) encapsulating 9-Phe for the treatment of brain edema post-ischemic stroke in mice. This system, named 9-Phe@T7-LNP, leveraged the brain-targeting ability of T7 (HAIYPRH) (Zong et al., 2014) and the strong biocompatibility of PEG-LNP (**Figure 6D**). It was found that 9-Phe@T7-LNP exhibited notable pharmacokinetic properties, slow systemic elimination, and good targeted accumulation in the ischemic hemisphere. Additionally, it effectively inhibited TRPM4 channels, reduced brain edema, and improved overall neurological function.

Protoporphyrin IX is a widely used sonosensitizer that can be connected to drug delivery systems to spatiotemporally control the delivery of drugs (Liu et al., 2024). Li et al. (2021) engineered a microglial (MG) platform decorated with platelet membranes (PMs) (PM-MG) to improve adherence to injured cerebral vessels and improve targeting. Ultrasound-responsive liposomes containing protoporphyrin IX and IL-4 (CPIL4) were attached to the PM-MG platform through a click reaction, forming PM-MG-CPIL4. Ultrasound irradiation promoted the release of IL-4 from encapsulated liposomes, leading to M2 polarization of microglia (Li et al., 2021b). PM-MG-CPIL4 not only demonstrated enhanced retention at the stroke site compared with that in non-decorated microglia, with significant adherence observed in injured mouse brains but also promoted neuroprotection and recovery through ultrasound-triggered IL-4 release.

Reconstituted lipid nanoparticles (rLNPs) represent advanced drug delivery systems, characterized by their high reproducibility and suitability for cell- and tissue-based applications. rLNPs retain the merits of conventional synthetic LNPs while incorporating naturally derived lipids. This feature allows them to preserve the characteristics of the original cell or tissue, which facilitates potential targeted delivery and homotypic interactions. Han et al. (2024) developed and evaluated brain-derived rLNPs (B-rLNPs) as a targeted vehicle for ischemic stroke. They found that, compared with liver-derived rLNPs, B-rLNPs exhibited improved targeting and accumulation in the ischemic brain region. Additionally, B-rLNPs displayed minimal cytotoxicity and hemolytic activity, suggesting a favorable safety profile for potential clinical applications. The authors further loaded B-rLNPs with the drug NBP and found that these LNPs had notable therapeutic efficacy against ischemic stroke, reducing infarct volume and improving neurological function. Although BBB integrity was not fully restored, B-rLNPs/NBP reduced BBB permeability compared to free NBP, indicating that it exerted a potential protective effect against BBB disruption (Han et al., 2024).

### Biomimetic nanoparticles

Cell membrane coating technology has recently been used in the manufacture of a variety of therapeutic nano-drug delivery systems, with promising results (Broughton et al., 2009). This technique not only enhances the biocompatibility of the NPs but also extends their circulation time and improves targeting efficacy *in vivo* (**Table 4**).

**Table 4:** Biomimetic NPs in ischemic stroke treatment

Biomimetic membranes	Biomimetic functions	Loaded drugs	Therapeutic mechanisms	References
Neutrophil membranes	Targeting inflammatory areas and crossing the BBB	FTY720	Reprogramming microglial from M1 phenotype to M2 phenotype	Zhao et al., 2024
		ResolvinD2	Decreasing neutrophil infiltration and cytokine levels induced inflammation	Dong et al., 2019
		Edaravone and SHp	Scavenging ROS, inhibiting caspase-3, enhancing Bcl-2 and suppressing Bax	Dong et al., 2024
4T1 cancer cells membranes	Crossing the BBB	Succinobucol	Scavenging ROS	He et al., 2021
Platelet membranes	Targeting thrombus and escaping immune clearance	Melanin NPs and tPA	Scavenging free radicals and improving blood flow restoration	Yu et al., 2022
Bacteria-derived outer membrane vesicles	Targeting neutrophil TLR	PGZ	Inhibiting NLRP3 inflammasomes and ferroptosis	Pan et al., 2023
Mesenchymal stem cell membranes	Targeting to SDF-1 via CXCR4 and specifically binding to VCAM-1 via VLA-4	ZL006 and Prussian blue NPs	Scavenging ROS and inhibiting the interaction of nNOS synthase with PSD-95	Zhang et al., 2024a
Embryonic stem cell extracellular vesicles	Modulating Tregs	Undescribed	Activating the TGF-β/Smad pathway for Tregs	Xia et al., 2021
M2 macrophages exosomes	Crossing BBB and inducing the polarization of M1 microglia to M2 phenotype	DNase 1	Inhibiting the formation of NETs and increasing M2 microglia	Wang et al., 2024

Bax: Bcl-2-associated X protein; Bcl-2: B-cell lymphoma 2; CXCR4: C-X-C motif chemokine receptor 4; FTY720: fingolimod; NLRP3: nucleotide oligomerization-like receptor protein 3; NPs: nanoparticles; PGZ: pioglitazone; PSD-95: postsynaptic density protein 95; ROS: reactive oxygen species; SDF-1: stromal cell-derived factor-1; SHp: stroke-homing peptides; TGF-β: transforming growth factor-beta; TLR: toll-like receptors; Tregs: regulatory T cells; VCAM-1: vascular cell adhesion molecule-1; VLA-4: very late antigen-4.

**Figure 7:**
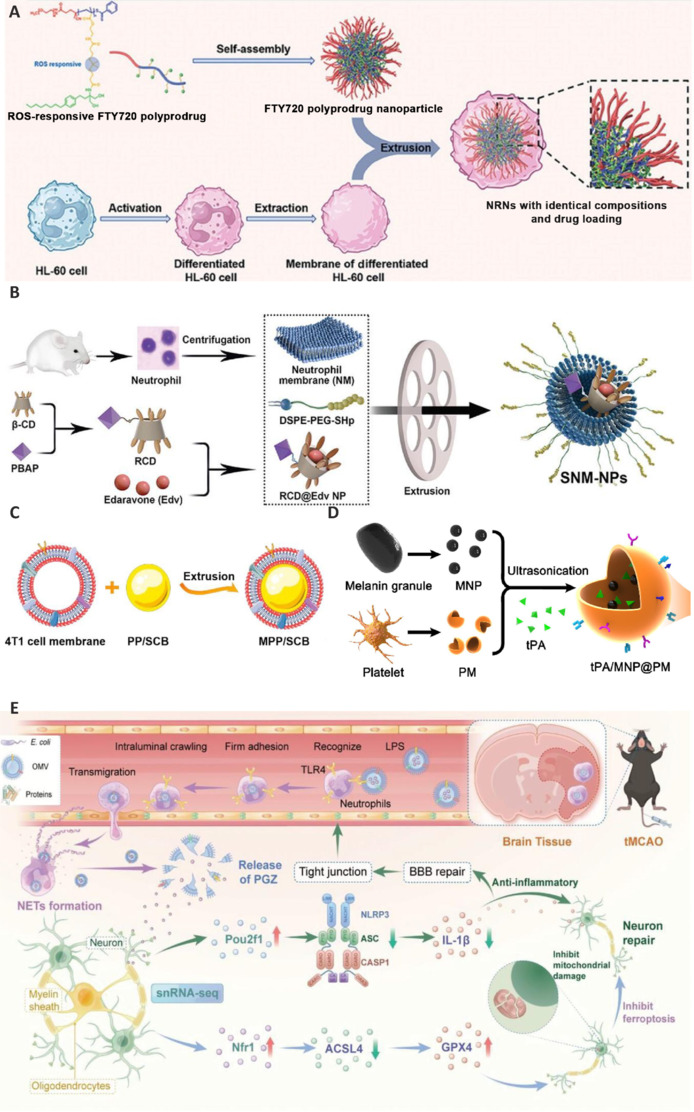
Biomimetic NPs for treating ischemic stroke. (A) Self-assembly of FTY720 polyprodrug NP coated with neutrophil membranes. Reproduced with permission from Zhao et al. (2024). Copyright © 2024 Wiley-VCH GmbH. (B) The synthesis process of SNM-NPs. Reproduced with permission from Dong et al. (2024). Copyright © 2023 Wiley-VCH GmbH. (C) PP/SCB NP coated with 4T1 cell membrane. Reproduced with permission from He et al. (2021). Copyright © 2021 American Chemical Society. (D) The fabrication of tPA/MNP@PM. Reproduced with permission from Yu et al. (2021). Copyright © 2021 Acta Materialia Inc. (E) OMV@PGZ hitchhiked on neutrophils across the BBB to the brain and released PGZ to treat ischemic stroke. Reproduced with permission from Pan et al. (2023). Copyright © 2023 Wiley-VCH GmbH. ACSL4: Acyl-CoA synthetase long-chain family member 4; ASC: apoptosis-associated speck-like protein; CASP1: caspase-1; E.coli: escherichia coli; FTY720: fingolimod hydrochloride; GPX4: glutathione peroxidase 4; HL-60: human promyelocytic leukemia; IL-1β: interleukin-1β; LPS: lipopolysaccharide; MNP: melanin NP; NETs: neutrophil extracellular traps; NLRP3: nucleotide oligomerization-like receptor protein 3; NP: nanoparticle; Nrf1: nuclear respiratory factor 1; OMV: outer-membrane vesicle; PBAP: (hydroxymethyl)phenylboronic acid pinacol ester; PEG-DSPE-SHp: polyethylene glycol-1,2-distearoyl-sn-glycero-3-phosphoethanolamine-stroke-homing peptides; PGZ: pioglitazone; PM: platelet membrane; Pou2f1: pou class 2 transcription factor 1; PP/SCB: pH-sensitive polymeric NPs of succinobucol; RCD: β-CD-PBAP; ROS: reactive oxygen species; TLR4: toll-like receptor 4; tMACO: transient middle cerebral artery occlusion; tPA: thrombolysis with tissue plasminogen activator; β-CD: β-cyclodextrins.

Neutrophils, immune cells known for their rapid response to inflammation, can sensitively detect increases in the levels of inflammatory cytokines through their receptors (Nishikimi et al., 2009; Adrover et al., 2019; Burn et al., 2021). Following detection, they migrate toward the site of injury. Neutrophil membrane-coated NPs acquire the antigenic properties and relevant membrane functionalities of the original cells, enabling them to home in on areas of inflammation as well as cross the BBB. Similarly, nanozymes coated with neutrophil-like cell membranes can selectively target damaged brain regions, and display an enhanced ability to traverse the BBB by adhering to inflamed brain microvascular endothelial cells through surface adhesion molecules. FTY720 is an FDA-approved anti-inflammatory drug with potential neuroprotective effects. The primary obstacle to its clinical application is how to efficiently deliver FTY720 across the BBB to brain lesions without causing severe cardiovascular side effects. Zhao et al. (2024) developed a neutrophil membrane-camouflaged prodrug nanosystem capable of delivering FTY720 to ischemic brain tissues and responding to ROS levels while minimizing side effects (**Figure 7A**). To achieve this, FTY720 monomers were polymerized and then wrapped with neutrophil membranes for targeted delivery. Compared to the free drug, this nanosystem achieved a 15.2-fold increase in FTY720 delivery to the ischemic brain, successfully promoting the polarization of microglia from a pro-inflammatory to an anti-inflammatory phenotype, and reducing neuroinflammation. Moreover, the targeted delivery reduced the cardiotoxicity and infection risks associated with high doses of FTY720. Resolvin D2 (RvD2) is a specialized pro-resolving mediator derived from eicosapentaenoic acid, an omega-3 fatty acid. It plays an important role in resolving inflammation and returning the body to a state of homeostasis after an inflammatory response (Serhan et al., 2014; Duvall and Levy, 2016; Moro et al., 2016; Balta et al., 2017). Dong et al. (2019) explored a therapeutic strategy involving the use of neutrophil membrane-derived nanovesicles to deliver RvD2 for the treatment of ischemic stroke. They demonstrated that these nanovesicles can target inflamed brain endothelium, reducing inflammation and promoting neuroprotection. The approach significantly decreased neutrophil infiltration and cytokine levels, while promoting the recovery of brain injury and improving neurological function (Dong et al., 2019). Stroke-homing peptides (SHp), identified through *in vivo* screening of a bacteriophage library, can specifically target apoptotic neural cells at the site of stroke (Lv et al., 2018). They were shown to exhibit a strong affinity for glutamate receptors present on the surface of neurons undergoing apoptosis induced by oxidative stress (Hong et al., 2008; Lv et al., 2018). Consequently, NPs decorated with SHp can be used to target apoptotic neurons in the cerebral ischemia site (Hong et al., 2008). Dong et al. (2024) designed a drug delivery system, SNM-NPs, in which ROS-responsive cyclodextrin and EDA were encapsulated in a neutrophil membrane coated with SHp (**Figure 7B**). SNM-NPs induced a 5.16-fold increase in BBB permeability in the presence of formyl-methionyl-leucyl-phenylalanine compared to controls. Notably, EDA and ROS-responsive cyclodextrin, which were delivered by SNM-NPs, effectively scavenged ROS, reduced inflammatory factor levels, and inhibited caspase-3 activity. SNM-NPs also upregulated the expression of B-cell-lymphoma-2 (Bcl-2) and suppressed that of Bcl-2-associated X (BAX), thereby inhibiting nerve cell apoptosis and promoting nerve repair (Dong et al., 2024).

Several tumor cells, such as breast cancer and melanoma cells, can cross the BBB and metastasize to the brain (Wang et al., 2020). CD138, expressed on the membranes of 4T1 cancer cells, can bind to CD31, extensively present on platelets, leukocytes, and endothelial cells, within blood vessels of the brain. This binding facilitates the migration of cancer cells across the BBB (Barbareschi et al., 2003; Chute et al., 2018; Sayyad et al., 2019). Additionally, vascular cell adhesion molecule 1, highly expressed on the surface of 4T1 cancer cells, strongly binds to very late antigen-4, a protein found on leukocytes (Calabresi et al., 1997; Vainio et al., 2019). He et al. (2021a) developed a biomimetic platform, MPP/SCB, involving the encapsulation of a succinobucol-loaded amphiphilic pH-sensitive polymer (PEG-PDPA) in a 4T1 cancer cell membrane, thus enhancing targeting to ischemic brain regions (**Figure 7C**). MPP/SCB effectively targeted cerebral ischemic lesions and reduced infarct volume by 69.9% in a rat model of tMCAO (He et al., 2021a).

As mentioned above, platelets possess an inherent ability to target injured vascular sites, creating a clot to prevent excessive bleeding. Platelet membranes exhibit thrombus-targeting capabilities and the capacity to evade immune clearance. The targeting and activation of platelets are closely associated with pathological changes at sites of infarction (Nieswandt et al., 2011). Yu et al. (2022) developed a biomimetic nanovesicle (tPA/MNP@PM, tMP), which combined tPA and melanin NPs (MNPs) within a platelet membrane via one-step ultrasonication. The biomimetic nanovesicles exhibited enhanced targeting to thrombotic sites owing to the inherent adhesive properties of the platelet membrane (**Figure 7D**). Meanwhile, the photothermal properties of the MNPs facilitated the controlled release of tPA in response to near-infrared irradiation. Accordingly, as the temperature increased, so did the thrombolytic efficacy of tPA. In addition, the released MNPs crossed the BBB and accumulated at cerebral ischemia sites, where they scavenged free radicals and reduced inflammatory and immunological injuries. In MCAO models, this treatment demonstrated significant advantages over conventional approaches, improving blood flow restoration, reducing infarct size, and mitigating tPA-induced cerebral hemorrhage (Yu et al., 2022).

EVs are membranous bilayer structures secreted by most cell types in eukaryotes, including fungi, as well as Gram-negative and Gram-positive bacteria (Yáñez-Mó et al., 2015; Théry et al., 2018). Bacteria-derived outer membrane vesicles (OMVs) contain LPS, which can be directly recognized by pattern recognition receptors. Pan et al. (2023) explored a novel therapeutic strategy involving OMVs that hitchhiked on neutrophils to deliver pioglitazone (PGZ) to the brain (**Figure 7E**). The results showed that OMV@PGZ successfully hitchhiked on neutrophils across the BBB to the brain, where they inhibited nucleotide oligomerization-like receptor protein 3 inflammasomes and ferroptosis, thereby alleviating oxidative stress and reperfusion injury. Furthermore, treatment with OMV@PGZ significantly reduced the cerebral infarction area in tMCAO mice. Moreover, through single-nucleus RNA sequencing (snRNA-seq), the authors identified two transcriptional regulators, POU domain class 2 transcription factor 1 (Pou2f1) and nuclear factor erythroid 2-related factor 1 (Nrf1), that played a role in neural repair. Additionally, OMV@PGZ treatment significantly restored the levels of subcluster 5 oligodendrocytes, which play a vital role in oxidative stress regulation and BBB repair (Pan et al., 2023).

Mesenchymal stem cells (MSCs) can express a diverse range of proteins (Wu et al., 2019; Zhong et al., 2024) and release therapeutic biomolecules to treat ischemic stroke (Lee et al., 2010). It has been demonstrated that exosomes derived from MSCs improve behavioral, neurological, and cognitive outcomes in animal models (Pathipati et al., 2021). MSC-derived exosomes were shown to enhance neurovascular reconstruction during recovery and facilitate the repair of neuronal circuits in various brain injury models. Zhang et al. (2024) designed a multifunctional nanoplatform (PB-006@MSC) for the co-delivery of the neuroprotectant ZL006 and Prussian blue NPs. This platform used MSC membrane biomimetics to treat oxidative stress and excitotoxicity (Mu et al., 2023). Treatment with PB-006@MSC resulted in a 4-fold increase in ZL006 accumulation at the ischemic penumbra compared to the control treatment, demonstrating that MSC membrane coating enhanced the homing capabilities of the platform. Besides, PB-006@MSC reduced cerebral infarct volume from 37.1% to 2.3%. The results of this study highlighted the ROS scavenging properties of PB-006@MSC, which alleviated oxidative stress in neuronal cells and improved overall neuroprotection in the ischemic environment.

Embryonic stem cell-EVs (ESC-EVs) have shown promising tissue regeneration capabilities (Khan et al., 2015). Xia et al. (2021) investigated the potential of ESC-EVs in improving ischemic brain damage through the modulation of regulatory T cells (Tregs), which play important roles in maintaining immune homeostasis and suppressing inflammation. They found that ESC-EVs induced a significant increase in Treg numbers. Proteomic analysis identified the presence of transforming growth factor-beta (TGF-β) and Smads (Smad2, Smad4) in ESC-EVs. When activated, the Smad/TGF-β pathway can promote Treg proliferation. These findings demonstrate that ESC-EV therapy may represent a promising approach for stroke treatment through immune modulation (Xia et al., 2021).

M1 microglia release inflammatory cytokines that can lead to tissue damage, whereas M2 microglia secrete anti-inflammatory cytokines, which exert neuroprotective effects. Exosomes from M2 macrophages (M2exo) facilitate M2 polarization by secreting anti-inflammatory cytokines. Wang et al. (2024) found that M2exo can serve as effective carriers for DNase 1, enhancing its delivery to the ischemic brain and promoting anti-inflammatory responses. This study reported the development of a nanoplatform (M2exo@DNase 1) via the encapsulation of DNase 1 in exosomes derived from IL-4-polarized M2 macrophages. M2exo@DNase 1 significantly inhibited the formation of NETs *in vitro*, resulting in a 38.6% reduction in NET formation within 15 minutes. Furthermore, real-time monitoring showed a significant recovery in cerebral blood flow, reaching 91.9% post-treatment. The therapeutic effects were corroborated by MRI imaging, which showed a 74% reduction in the brain lesional area at 28 days. Overall, M2exo@DNase 1 not only inhibited NET formation but also increased the proportion of anti-inflammatory M2 microglia, highlighting its potential as a therapeutic agent for stroke.

### Prospects and challenges associated with the clinical translation of nano-based drug delivery systems

Several clinical trials are currently exploring the use of liposomes to enhance BBB penetration for small molecules. These liposomes have demonstrated efficacy in treating brain tumors (Cai et al., 2024) and show promising prospects for ischemic stroke treatment. NP-based drug delivery systems are gradually advancing personalized medicine by improving drug delivery efficiency and promoting the development of novel therapeutic approaches. However, the progression of NP-based drug delivery systems from preclinical research to clinical application faces several challenges that must be addressed. Foremost among these challenges is the potential neurotoxic effects of NP-based drug delivery systems, which require careful evaluation. Metal-based NPs not only damage the BBB but also cause astrocyte swelling, resulting in neuronal degeneration (Tang et al., 2009). In contrast, biomimetic NPs, which inherit characteristics of natural cells, are generally considered to have relatively low toxicity and immunogenicity. Nonetheless, research has shown that platelet cells can contain and release substances capable of causing neuronal dysfunction (Joseph et al., 1992). Similarly, hemoglobin derived from red blood cells exhibits neurotoxicity, leading to neuronal death after 24–48 hours of exposure, although no significant toxic effects were observed during shorter exposure periods (1–2 hours) (Regan and Panter, 1993). Additionally, tumor cell-derived membranes have been used as coatings for nanocarriers (Wang et al., 2020). However, concerns remain regarding the incomplete removal of genetic material and nuclei from these tumor cells, which could introduce carcinogenic risks when using tumor cell membrane-coated NPs (Xu et al., 2020). Consequently, thorough safety assessments are essential for minimizing risks and ensuring the clinical viability of such advanced drug delivery platforms. Additional challenges in clinical translation include regulatory compliance, feasibility of large-scale manufacturing, consistency between batches, long-term storage stability, and the ability to accurately predict human responses based on animal models. Overcoming these obstacles and successfully translating promising NP technologies into effective therapeutic solutions for patients requires a multidisciplinary approach involving scientists, engineers, clinicians, and regulatory authorities.

## Limitations

The limitations of this review arise primarily from its constrained length and the limited scope of the evaluated literature, which spans only 5 years (September 2019 to September 2024). The review primarily focuses on the application of neuroprotective agents in combination with nanodelivery systems for the treatment of ischemic stroke in detriment to articles related to physiology or biology. Additionally, some underlying mechanisms were not thoroughly investigated in the existing literature.

## Conclusion and Perspectives

Ischemic stroke is a significant cause of mortality and severe disability worldwide. Thrombolysis is currently the most direct and effective clinical treatment for ischemic stroke; however, its application is limited by a narrow therapeutic time window. Furthermore, the restoration of blood flow following a stroke can trigger a cascade of detrimental processes, known as ischemia-reperfusion injury, that include excitotoxicity, neuroinflammation, and oxidative stress, all of which contribute to the exacerbation of brain damage. This indicates the urgent need to further explore the pathogenesis of ischemic stroke and develop potential neuroprotective interventions.

In addition to vascular recanalization, neuroprotective therapy plays a crucial role in stroke treatment. Its aim is to reduce ROS levels at the ischemic site, inhibit neuronal cell death, and promote regeneration. The combination of neuroprotective agents with thrombolytic therapy has shown considerable promise in improving the treatment of ischemic stroke by mitigating the effects of ischemia-reperfusion injury. However, traditional neuroprotective agents often face significant limitations, including high toxicity, low water solubility, and limited stability. Notably, advancements in nanotechnology have revolutionized drug delivery through nano-based systems, thus addressing these challenges and enhancing therapeutic efficacy. Nanodrugs offer several advantages over conventional drugs, including improved penetration of the BBB, extended circulation time in the body, enhanced therapeutic effects, significant drug-loading capacity, and customizable properties. These attributes make nanodrugs promising candidates for ischemic stroke research. Moreover, findings from studies on nano-based drug delivery systems have underscored the importance of a multidisciplinary approach in addressing the complexities of nerve regeneration. Integrating insights from neuroscience, materials science, and pharmacology can facilitate the development of effective therapeutic strategies.

In this review, we summarized the pathophysiological mechanisms and related drugs for ischemic stroke, highlighted recent advancements in NP research, and presented novel perspectives for future studies. Specifically, inorganic NPs, such as metallic oxide NPs, can modulate intracellular oxidative stress and reduce inflammation by altering the charge and valence states of metal ions. Polymeric NPs, including those made from PEG and PLGA, have been shown to significantly improve drug solubility, stability, and transmembrane transport, thereby enhancing drug biocompatibility and bioavailability. LNPs, constructed from natural biodegradable and biocompatible amphiphiles such as fatty acids and phospholipids, can be surface-functionalized with targeting ligands to improve targeting precision. Biomimetic NPs, including those coated with cell membranes or EVs, can cross the BBB and selectively target ischemic regions while maintaining excellent biocompatibility and bioavailability. In conclusion, the unique properties of NPs—such as enhanced targeting, stability, and transport capabilities—make them superior to traditional small-molecule drugs, offering significant potential for advanced therapeutic applications. NPs can improve drug pharmacokinetics and targeting to lesion areas, thereby reducing adverse effects and complications. Recent advancements in nanotechnology have led to the development of NP-supported neuroprotective therapies, which show substantial promise in treating ischemic stroke.

Although the neuroprotective therapies supported by the NPs mentioned above have shown promising results in cell and animal studies, their clinical application still faces significant challenges. Currently, the industrialization of nanomedicine is hindered by high costs and low stability, while the safety implications arising from the presence of NPs in the brain and the rest of the nervous system require long-term investigation. In this context, we propose considerations for the clinical translation of NP-associated treatments and emphasize the challenges inherent in their application for the treatment of ischemic stroke.

From a clinical perspective, there is a significant gap between laboratory findings and nanomedicine application in clinical practice. This review aimed to bridge that gap, serving as a catalyst for further research and ultimately enhancing the prospects for patients who have experienced ischemic stroke. It introduces nano-based drug delivery systems that have the potential to overcome the limitations of traditional therapies, such as off-target effects and poor BBB penetration. The use of NP-based therapies can improve treatment efficacy while reducing complications. Additionally, the review highlighted how NP-based therapies can be integrated with existing interventions, such as thrombolysis, to provide a comprehensive approach to stroke management. Moving forward, several directions can be proposed to advance the field of nanotechnology-based therapies for ischemic stroke and nerve regeneration. First, future research should focus on elucidating the mechanisms by which NPs influence nerve regeneration. This includes investigating how they interact with neuronal cells, glial cells, and the extracellular matrix to promote axonal growth, synaptic remodeling, and neurogenesis. Understanding these interactions will facilitate the design of NPs that specifically target regenerative pathways. Second, we propose the development of multifunctional NPs that combine neuroprotective, anti-inflammatory, and regenerative properties. For instance, NPs could be designed to release growth factors or stem cell-derived exosomes in addition to delivering neuroprotective agents, thereby providing a more holistic approach to stroke therapy. Lastly, although NPs have shown promise in preclinical studies, their long-term safety and efficacy in humans remain uncertain. Future research should prioritize conducting rigorous preclinical and clinical trials to evaluate the potential toxicity, immunogenicity, and pharmacokinetics of NPs in large model animals or even human patients. These studies will be critical for ensuring the safe and effective translation of nanotechnology-based therapies.

Nanotechnology-based drug delivery systems hold great potential for treating ischemic stroke. Understanding the connection between neuroprotective NPs, the microenvironment of ischemic stroke, molecular signaling pathways, and the underlying pathophysiology is crucial for the clinical application of these systems.

This review holds significant value for both researchers and clinicians working in the fields of ischemic stroke and nerve regeneration. By examining the latest advancements in nanotechnology-based drug delivery systems, it outlines a roadmap to guide the development of more effective and targeted therapies for ischemic stroke. Furthermore, the review underscores the importance of understanding the interactions between NPs and the brain microenvironment. For example, it discusses how NPs can modulate key processes involved in nerve regeneration, such as neuronal survival, axonal growth, synaptic plasticity, and angiogenesis. By elucidating these mechanisms, the review provides a foundation for designing NPs that not only deliver drugs to the brain but also actively support its intrinsic repair processes. The review emphasizes the particular importance of promoting nerve regeneration after stroke, as existing therapies primarily focus on minimizing neuronal damage during the acute phase of the condition. There is a vital need for long-term strategies that promote neuronal repair and functional recovery.

In summary, this review comprehensively summarized the contributions of previous studies, identified existing challenges, and proposed future directions for stroke treatment. The clinical translation and widespread adoption of these strategies are ongoing. We anticipate that continued research and innovation will unlock the full potential of nanotechnology in supporting neuronal repair and improving outcomes for stroke patients.

## Data Availability

*Not applicable*.
